# Artificial Intelligence for Management of Major Depression: Initial Design, Progress, and Research Plans

**DOI:** 10.31083/AP44608

**Published:** 2025-07-08

**Authors:** Farrokh Alemi, Janusz Wojtusiak, Aneel Ursani, K. Pierre Eklou, Kevin Lybarger

**Affiliations:** ^1^Health Administration and Policy, George Mason University, Fairfax, VA 22030, USA; ^2^Klinic Inc., Seattle, WA 98101, USA; ^3^School of Nursing, George Mason University, Fairfax, VA 22030, USA; ^4^Information Sciences and Technology, George Mason University, Fairfax, VA 22030, USA

**Keywords:** antidepressant-resistant depression, antidepressant, depression, major depressive disorder

## Abstract

**Background:**

Herein, we report on the initial development, progress, and future plans for an autonomous artificial intelligence (AI) system designed to manage major depressive disorder (MDD). The system is a web-based, patient-facing conversational AI that collects medical history, provides presumed diagnosis, recommends treatment, and coordinates care for patients with MDD.

**Methods:**

The system includes seven components, five of which are complete and two are in development. The first component is the AI’s knowledgebase, which was constructed using Least Absolute Shrinkage and Selection Operator (LASSO) logistic regression to analyze extensive patient medical histories and identify factors influencing response to antidepressants. The second component is a series of adjustments to the knowledgebase designed to correct algorithm bias in patient subgroups. The third component is a conversational Large Language Model (LLM) that efficiently gathers patients’ medical histories. The fourth component is a dialogue management system that minimizes digressions in the LLM conversations, using a topic network statistically derived from the AI’s own knowledgebase. The fifth component is planned to enable real-time, human-in-the-loop monitoring. The sixth component is an existing analytical, non-generative module that provides and explains treatment advice. The seventh component is planned to coordinate care with clinicians via closed-loop referrals.

**Results:**

In component 1, the AI’s knowledgebase correctly predicted 69.2% to 78.5% of the variation in response to 15 oral antidepressants. Patients treated by AI-concordant clinicians were 17.5% more likely to benefit from their treatment than patients of AI-discordant clinicians. In component 2, the use of the system required adjustments to improve accuracy for predicting the responses of African Americans to four antidepressants and no adjustments were required for the remaining 10 antidepressants. In component 3, the conversational intake efficiently covered 1499 relevant medical history events (including 700 diagnoses, 550 medications, 151 procedures, and 98 prior antidepressant responses). In the fourth component, the dialogue management system was effective in maintaining a long dialogue with many turns in the conversation. In the sixth component, the advice system was able to rely exclusively on pre-set text. An online ad campaign attracted 1536 residents of Virginia to use the advice system. Initially, a focus group of clinicians was skeptical of the value of the advice system and requested more prospective studies before they would implement the system in their clinics. When the system was redesigned to advise patients at home, clinicians were willing to receive referrals from the system and discuss the advice of the system with their patients.

**Conclusions:**

Further research is needed to refine and evaluate the system. We outline our plans for a prospective randomized trial to assess the system’s impact on prescription patterns and patient outcomes.

## Main Points

1. *Antidepressant Knowledgebase:* This work describes a methodology for retrospective analysis of extensive patient medical histories to identify the most effective antidepressants tailored to complex and diverse patient profiles. It presents an approach for assessing and mitigating algorithmic biases in antidepressant recommendations, with demonstrated improvements for African American populations.

2. *Preliminary Conversational Agent for Antidepressant Recommendations:* This manuscript presents the development and evaluation of a conversational AI-driven decision aid designed to efficiently collect patient medical histories and provide personalized antidepressant recommendations based on a comprehensive knowledgebase. This AI system uses a novel dialogue management system that leverages the antidepressant knowledgebase to achieve goal-oriented patient interactions.

3. *Human-in-the-Loop Monitoring:* We present a safety-focused framework for deploying the conversational agent within a human-in-the-loop setting, enabling real-time monitoring of patient interactions to mitigate risks, particularly around suicide detection and prevention.

4. *Care Coordination:* The paper outlines an approach for integrating the AI system into clinical care, emphasizing closed-loop referrals to ensure that recommendations are actionable, seamlessly delivered, and aligned with clinicians’ workflows.

## 1. Introduction

Major depressive disorder (MDD) is a prevalent and debilitating mental health condition that requires effective, personalized treatment strategies. Herein, we describe the development, current progress, and future plans for an artificial intelligence (AI)-driven decision aid aimed at improving the treatment of moderate to severe MDD through tailored antidepressant recommendations. The proposed system assumes that patients have already been diagnosed with MDD by a clinician or another AI system, focusing explicitly on optimizing treatment decisions and improving remission rates. Recent advancements in Large Language Models (LLMs) have raised the potential for AI to augment clinical activities by enabling coherent, natural language conversations tailored to health care needs. LLMs demonstrate the ability to engage in natural-language conversations [1] tailored to various healthcare needs [2]. These models can use empathetic tones and customize content for different audiences [3]. Natural language understanding capabilities allow LLMs to follow instructions, answer medical questions [4], and engage in short therapeutic conversations [5]. To date, LLM applications in healthcare have primarily focused on workflow efficiency and removing administrative burdens, with limited emphasis on enhancing clinical decision-making [6, 7].

LLMs face several challenges that hinder their direct application in healthcare, including behavioral health. These challenges include generating inaccurate but convincing information (“hallucinations”), topic drift during extended conversations, and the risk of culturally inappropriate or emotionally insensitive responses [8]. Ensuring patient safety is of paramount concern, especially in cases where patients may be at risk of suicide. Media reports have highlighted instances of AI systems providing harmful or inappropriate responses [9], further underscoring the need for robust safeguards in behavioral health applications. Even though AI can exhibit greater empathy than clinicians in brief questions and answers [10], AI-systems may not succeed in establishing the therapeutic bond necessary for mental health treatment [11].

Prescribing effective antidepressants is inherently complex due to the large number of available options (more than 20 antidepressants and many possible combinations), the limited pragmatic research that addresses patients’ comorbidities [12], and the limited availability of reports on negative findings [13]. Additionally, genetic profiling and simple rules-based approaches have shown limited utility in tailoring treatments to individual needs [14]. To address these challenges, we propose an AI system that leverages a network of interrelated predictive models that analyze patient medical history to predict the probability of response to various antidepressants, enabling patients and clinicians to make evidence-based decisions. Previous decision aids for antidepressant prescribing have emphasized improving patient satisfaction, shared decision-making, and medical adherence, with limited focus on remission rates. For example, Aoki *et al*. [15] developed a mixed-methods questionnaire based on systematic review and meta-analyses but did not focus on improving remission rates, and Abousheishaa *et al*. [16] developed a prototype based on a literature review and focused on assessing patient and provider perspectives, not remission rates. While there is evidence that depression management aids can improve patient-provider exchanges, there is limited evidence that they improve treatment outcomes [17]. The AI system described herein prioritizes treatment outcomes by focusing explicitly on increasing remission rates through tailored recommendations.

While existing guidelines, such as those from Texas [18] and Canadian [19] frameworks, provide general guidance on antidepressant prescription, their impact on patient outcomes is unclear [20]. For instance, randomized trials of the Texas guidelines showed no significant differences in remission rates to care-as-usual groups, except after statistical adjustments for baseline characteristics [21]. These guidelines lack specificity in addressing comorbid conditions, leaving clinicians without clear instructions for individualized treatment.

This report focuses on the development and evaluation of an AI-driven decision aid designed to enhance the treatment of moderate to severe MDD. The system assumes that patients have already been diagnosed with MDD, either by a clinician or an AI system, and aims to optimize treatment decisions through tailored antidepressant recommendations. Unlike existing tools that primarily support shared decision-making, this system prioritizes improving remission rates by leveraging predictive analytics and conversational AI.

## 2. Methods

The proposed AI system is a web-based, evidence-based, independent, patient-facing platform that can be used outside of traditional clinical workflows for the management of MDD. The system has seven components: (1) a knowledgebase derived from massive pragmatic data; (2) a component to remove algorithm bias in subgroups of patients; (3) a conversational LLM that collects medical history; (4) a dialogue management component that reduces digression; (5) a real-time human-in-the-loop system that monitors the AI; (6) a probabilistic analytical, non-generative, component that identifies and recommends optimal treatment; and (7) a closed-loop referral and follow-up component. The referral and human-in-the-loop systems are under construction, while viable prototypes of the other components exist and are undergoing component-by-component testing. The methods and preliminary results of this testing are summarized in this report.

### 2.1 Methods of Component 1: Organization of the Knowledgebase

The knowledgebase of the AI system, which identifies highly tailored antidepressant recommendations, was developed as part of the first large-scale study of antidepressant effectiveness post- Food and Drug Administration (FDA) release [22]. This study was a retrospective, observational, cohort study. The cohort was identified using claims data available through OptumLabs and included patients from all states within the United States of America. The analysis focused on the experiences of 3,678,082 patients with MDD treated with 10,221,145 prescriptions of antidepressants (counting different medications not different doses). Separate Least Absolute Shrinkage and Selection Operator (LASSO) regressions were performed to assess responses to the most common antidepressants including desvenlafaxine, doxepin, amitriptyline, bupropion, citalopram, duloxetine, escitalopram, fluoxetine, mirtazapine, nortriptyline, paroxetine, sertraline, trazadone, and venlafaxine. In addition, we defined a catch-all treatment category labeled as “other”, which included other less common antidepressants or a combination of two antidepressants. The pattern of use of the most common antidepressants over the study period are provided in Table 1 [23].

**Table 1.** **Frequency of treatments among episodes of major depressive disorder**.

	N	%
Sertraline	1,268,882	12.41
Escitalopram	1,074,882	10.52
Citalopram	931,213	9.11
Bupropion	912,409	8.93
Fluoxetine	893,127	8.74
Venlafaxine	540,360	5.29
Paroxetine	482,638	4.72
Trazodone	468,852	4.59
Duloxetine	448,276	4.39
Amitriptyline	276,302	2.70
Mirtazapine	181,744	1.78
Bupropion & Escitalopram	110,282	1.08
Sertraline & Trazodone	107,508	1.05
Bupropion & Sertraline	103,866	1.02
Bupropion & Fluoxetine	95,455	0.93
Nortriptyline	91,340	0.89
Bupropion & Citalopram	86,108	0.84
Fluoxetine & Trazodone	83,578	0.82
Escitalopram & Trazodone	82,857	0.81
Citalopram & Trazodone	82,412	0.81
Desvenlafaxine	80,573	0.79
Bupropion & Trazodone	76,985	0.75
Trazodone & Venlafaxine	68,999	0.68
Duloxetine & Trazodone	62,092	0.61
Bupropion & Venlafaxine	55,449	0.54
Doxepin	54,042	0.53
Bupropion & Duloxetine	47,801	0.47
Pramipexole	43,782	0.43
Paroxetine & Trazodone	40,673	0.40

In these regression analyses, the dependent variable was the patient-reported remission, which was not available in the claims data. A surrogate measure was needed. We relied on premature abandoning of antidepressants within the first 10 weeks of prescription of the antidepressant. Patients may abandon their prescribed antidepressants for a variety of reasons, including ineffective treatment or treatment with unacceptable side effects. No matter what the underlying reason for discontinuation, abandonment indicates an incorrect treatment choice. Continued use of an antidepressant does not always indicate that it is effective, as some patients stay with their partially effective treatment. To clarify the relationship between premature discontinuation and self-reported remission of depression symptoms, Alemi and colleagues re-examined data from the STAR*D study (https://www.nimh.nih.gov/funding/clinical-research/practical/stard). In this database, both patient-reported remission of depression symptoms and patterns of discontinuation of antidepressants are available. Their study showed that premature discontinuation was nearly perfectly (c-statistic = 0.93) associated with self-reported lack of remission of depression symptoms [24]. In 10,221,145 episodes, within the first 100 days of the start of the episode, 4,729,372 (46.3%) patients continued their treatment, 1,306,338 (12.8%) switched to another medication, 3,586,156 (35.1%) discontinued their medication, and 599,279 (5.9%) augmented their treatment.

The response to each antidepressant was predicted from 40,784 medical history events. Each of these events were included in the analysis as a binary variable having binomial distribution. The medical history events included illness history, prior experience with antidepressants, participation in psychotherapy, evaluation for psychiatric hospitalization, and current medications besides antidepressants. In examining responses to prior antidepressants, prior responses to individual antidepressants were examined and not prior responses to antidepressant types. In examining current medications besides antidepressants, each medication was considered separately. Medications were not combined into broad categories.

The SAFE rule was used to limit the predictors to the 1000 most relevant variables [25]. The SAFE rule is a procedure for discarding variables that have no impact by themselves on response to antidepressants. LASSO regressions were used to select the variables that were predictive of the response to the antidepressant from among the 1000 most relevant medical history events. The regressions were repeated in 40 randomly selected subsets of data to ensure that the findings were robust.

### 2.2 Methods of Component 2: Removing Algorithmic Bias

To address algorithmic bias, we used the All of Us database (https://allofus.nih.gov/), a resource organized by the National Institutes of Health that includes electronic health record (EHR) data from a diverse cohort of participants. This database is designed to over-sample minority populations, making it particularly suitable for analyzing health disparities [26]. As of writing, more than 781,000 participants have consented to participate, with over 400,000 having uploaded their EHR records. Among these, 250,500 of consented participants had at least one mention of depression, excluding bipolar depression. The analysis was done on the same set of antidepressants examined in component 1. Because All of Us data did not include patient-reported remission of symptoms of depression, we used discontinuation of the antidepressant as a marker for lack of response (see discussion of outcome variable in component 1).

We evaluated algorithmic bias in African Americans, and plan to also examine it in Hispanic, Asian, and other ethnic subgroups. LASSO regression models were developed for the common oral antidepressants (desvenlafaxine, doxepin, amitriptyline, bupropion, citalopram, duloxetine, escitalopram, fluoxetine, mirtazapine, nortriptyline, paroxetine, sertraline, trazadone, venlafaxine), and a catch-all category that was named “other”. The dependent variable was the continuation of prescribed antidepressant for 10 weeks, as discussed in the Methods of Component 1. The independent variables were also the same as discussed in the Methods of Component 1. For each antidepressant two regression models were derived from the data. The two regressions differed in the independent variables used to predict response to the antidepressants.

(1) In the regression labeled “general population model”, the independent variables were derived from the general population. The general population model was developed in component 1, using the OptumLabs data.

(2) In the regression labeled “Population Specific”, the independent variables were statistically derived from examining predictors of response to antidepressants within the African American population. The population specific model was developed using All of Us data. We focused on African Americans because this population has historically faced disparities in mental health treatment, including lower rates of appropriate antidepressant prescriptions and poorer treatment outcomes.

The accuracy of the two models were reported using McFadden’s R^2^. If the inclusion of new variables significantly improved the model’s ability to explain variations in response to antidepressants compared with the general model, then the AI’s knowledgebase was updated to reflect these findings. This adaptive approach ensures that the system can evolve to address disparities in antidepressant treatment outcomes across diverse populations.

### 2.3 Methods of Component 3: Conversational Intake

The AI analytical models need information on 1499 relevant medical history events, including 700 diagnoses (using international classification of disease codes), 550 medications (using HEDIS National Drug Codes, without counting dose differences), 151 procedures (using select Current Procedural Terminology codes), and 98 prior antidepressant responses (using prior-year response to common antidepressants). We developed two intake strategies: (1) a survey tool that collected the status of only the major predictors of response, which used multiple choice, close-ended questions; and (2) a conversational intake tool, which used LLMs and asked open-ended questions. The multiple-choice survey intake system used event tree analysis [27] to dynamically limit and identify the next most informative question based on previous responses. This approach mirrors the way clinicians process medical histories [28], focusing on the most pertinent information given the patient’s context, often leaving some events unverified due to time constraints [29]. For example, male patients are not asked if they are pregnant. To streamline the intake process, the survey method also focused on events that have the largest impact on treatment selection. In the AI’s knowledgebase, we examined 10,221,145 antidepressant treatments. We constructed combinations of features and examined the responses of patients within combinations of the most important medical history features/variables. A total of 16,770 unique combinations of the most important features were identified that described at least 100 patients. These unique combinations of the variables were used to guide the survey method in asking about relevant medical history. The survey began with questions about gender, age, and antidepressant history. It used this information to exclude combinations that were no longer of interest, leveraging information gain theory to optimize subsequent questions. The procedure simplified the average interview to fewer than 13 questions.

The conversational component asks open-ended questions and patients provide natural language responses. Patients may provide ambiguous or relevant responses, contradict previous responses, ask clarifying questions, or change topics, etc. For example, in response to “What is your sex?” the patient may say, “I have changed my gender”, “What do you mean by gender?”, “I am a 65-year-old female who has already tried citalopram”, or “I want to talk about a movie”. Patients are allowed flexibility in responding. The LLM interprets patient responses, including the identification of relevant medical history, if the response is within context, and generates natural language replies, including questions soliciting the next relevant medical history information.

The AI’s knowledgebase, organized in component 1, included many medical history events. A conversational collection of the medical history is preferred over survey methods because, in conversations, respondents do not need to list events that have not occurred and can focus on recall of events that have occurred. In contrast, in multiple choice surveys, the respondent answers both the events that have and have not occurred. In surveys, the decision aid must ask about each item, which results in a relatively long survey, even when earlier responses are used to rule out certain questions. One could use the ontology of diseases, mediations, and procedures to ask about broad categories and thus reduce the number of events that need to be verified. However, patients may not be aware of how diseases, medications, and procedures are classified, thus undermining efforts that rely on broad categories. In contrast, a conversational intake asks patients to recall only the events in their own history. This makes conversational intake more efficient than surveys.

One problem in the use of conversations in collecting medical history of depressed patients is that the patient may be suicidal. It is important for the AI system to recognize suicidal patients. Prior research has identified suicide risk in conversations [30]. LLMs can be used to monitor the dialogue for these known risk factors for suicide. Our AI system included the following prompt for recognizing the risk factors for suicide:

“Examine the patients’ responses to your questions to see if any of the following 14 risk factors for suicide are present: (1) active suicidal ideation, including expression of taking one’s life using a particular method and with a plan for when and how to do it; (2) passive suicidal ideation, including wishing to be dead without a specific plan; (3) history of suicidal behavior, including report of suicide attempt, interrupted suicide, or emergency room visits for suicide attempt; (4) non-suicidal or non-life-threatening self-injury; (5) thwarted belongingness, including rejection by a sexual partner; burden to others, including negative self-worth, no meaningful work, no caring for a child or adult, expression of “life would be easier without me”, and hopelessness; (6) persistent intolerable pain; acute exacerbation of mental illnesses, such as lack of compliance with psychiatric medications, sudden cessation of antidepressant use, report of new psychotic experiences, report of new mixed-state episode of bipolar depression, or report of untreated symptoms of depression; (7) new episodes of eating disorder or borderline/antisocial personality disorder; (8) preparatory suicidal actions, including report of new access to means of suicide such as guns or giving away cherished belongings/items; (9) significant and severe lack of sleep, including reports of nightmares and lack of Rapid Eye Movement (REM) cycle sleep; (10) adverse life events, such as report of death in the family, non-suicidal self-injury, recent suicide attempt among friends/school mates, or new diagnosis of a terminal or incurable disease; (11) report of victimization, including new encounters with persons responsible for sexual abuse of the patient, physical punishment, for example, parental punishment of teenagers, or physical peer victimization and bullying; (12) new indications of poor quality of attachment to parents or nuclear family; (13) sexual or gender confusion, including regrets for new sexual experimentation and “online outing” of sexual preferences; and (14) increase in recklessness or impulsivity including illicit substance use”.

### 2.4 Methods of Component 4: Dialogue Management System

In long conversations, such as multi-turn medical history intake, it is important to use a dialogue management system to keep the conversation on track. The Dialogue Manager decides whether to stay on the current topic (allowing for digressions) or transition to the next topic. If it needs to stay on the current topic, the Dialogue Management component does not change the context and the prompt to the LLM. If change is needed, then the Dialogue Management component changes instruction to the LLM. To make these decisions, this component needs to know what the relevant allowed digressions are. This is accomplished through creation of a Topic Network.

**Fig. 1.**
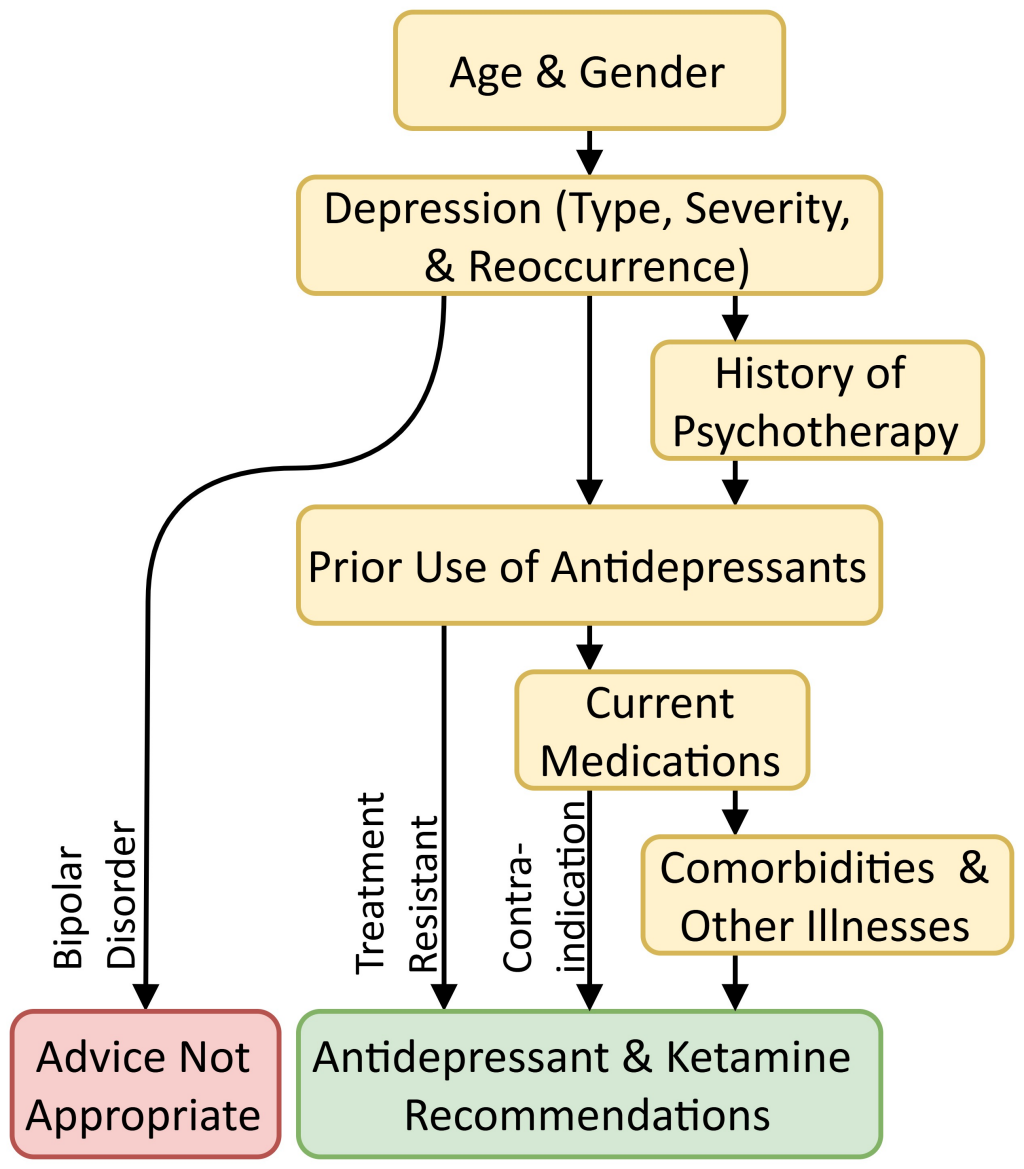
**Broad topic transitions**.

The Topic Network is a directed acyclical graph statistically derived from the knowledgebase of the AI system, in our case from the OptumLabs database (https://labs.optum.com/), using procedures described elsewhere [31]. Previous dialogue management research focused on Neural Networks [32]. We implement a Causal Network based on directed acyclical graphs that set priorities for the sequence in which topics should be processed. Fig. 1 shows an illustrative simplified set of topic categories; however, the actual Topic Network is more granular and cannot be presented here.

### 2.5 Methods of Component 5: Human-in-the-Loop Monitor

In real-world testing and deployment of the proposed system, we envision the use of human-in-the-loop monitoring to increase patient safety. Below is our proposed human-in-the in-loop monitoring approach. At each turn in the conversation, in real-time, the dialogue management system will send both the patient’s and the LLM’s deidentified and encrypted exchanges to a trained volunteer observer. When the monitor is not available, the AI system will be temporarily closed. If the AI or the human monitor detects a risk or threat of suicide, using the contact information provided by the patient prior to the use of the AI system, the system engages a family member [33], significant friend [34], or the patient’s mental health clinician to monitor the patient’s interactions with the system. The system cannot proceed unless a trained monitor is available. When the monitor signs in, the AI system describes the situation and trains the monitor regarding what to watch for. The monitor can decide if the patient can continue with the interview. If suicide is imminent, the monitor and the AI system encourage the patient to contact a suicide crisis line. If the interview is stopped or after the interview ends, the monitor is encouraged to help the patient to seek mental health care and organize a “safety plan”. As per consent approved prior to the start of the interview, the monitor can also initiate a call for help. In addition to suicide prevention, if the AI system makes inappropriate comments or discusses irrelevant topics, the monitor can instruct the AI system to correct itself and re-center the conversation on the medical history intake task.

There are two reasons for why we think the proposed human-in-the-loop system is scalable: (1) many components of the suicide detection mechanism are automated and therefore the need for human action is limited to individuals at some level of elevation of risk, and (2) the users of the system are asked to suggest a third-party monitor. These monitors are trained by the system prior to the use of the system. We have not yet finalized the training but an LLM-based system could briefly train monitors to detect inappropriate system replies and suicide risk factors. For patients who cannot or do not want to suggest monitors, we plan to rely on volunteers, typically clinicians in training in need of more hours of contact with real patients. Whether the training of monitors is effective and whether third-party monitors are widely available is an empirical issue that should be tested in future studies.

### 2.6 Methods of Component 6: Advice System

The advice system used the regressions in the knowledgebase of the AI system and the patient’s medical history collected by the LLM to predict the probability of response to every common antidepressant. For example, if the client was engaged in psychotherapy, then these regressions predicted the probability of a response to a specific antidepressant for clients who used antidepressants in conjunction with psychotherapy. If the client had a relevant sleep disorder or a specific mental health problem, then the regressions predicted responses for these types of patients. In addition, we predicted the medical history events that were not reported by the LLM through a separate set of equations used for assessing missing values. This system of linked regression equations is considered a network or structured equation model, where each regression identifies the variables that precede and are associated with one node in the network. One regression predicts the value of the independent variable (medical history event) in another set of regressions, which then predicts the response to an antidepressant.

In our design, the LLM does not “generate” advice used in the recommendation to the client using next-word probabilities, as is characteristic of LLMs. The recommendation is delivered through pre-set, non-generative text. Patients cannot interact with the advice component; the system refers patients to several existing, peer-reviewed, patient-facing, MedlinePlus websites that explain the benefits and harms of a specific recommended medication. Machine-generated text is only used during the intake interview, within which the potential harm to the patient is limited. Digression from the tasks is monitored by both the dialogue management system and, in the future, human monitors.

### 2.7 Methods of Component 7: Coordination of Care

The plan is for the AI system to alert clinicians through their patients. The patient receives the information first, and the patient is then encouraged to bring the advice to their clinician and discuss prescription changes. A prescription change is only made by the primary mental health care provider and only after a visit. If the patient does not have a primary mental health clinician, they are referred to a new clinician participating in our system. Our policy to inform the patient first is contrary to historical practices in computer-facilitated care, where clinicians are often informed first through EHR alerts. Our policy is similar to patients’ use of web-based calculators (e.g., [35]), albeit our system is more complex and interacts with the patient in natural language.

AI conversations could be time-consuming, especially if the patient asks clarifying questions. Long intakes can be disruptive to the clinical processes. By completing medical history at home, the long intake process is not a burden to the participating clinics. In addition, many clinicians are experiencing “alert fatigue” and they are turning off computer alerts, undermining the effectiveness of point-of-care procedures [36, 37]. By informing the clinician through the patient, the AI system may enhance the effectiveness of point-of-care alerts. The system educates the patient and may encourage them to actively participate in treatment decisions. Most patients with affective disorders want to participate in treatment decisions [38]; minority patients are particularly interested in active participation [39]. The clinicians may also prefer to directly hear from the patients about their concerns than to receive a computer alert [40].

The proposed system plans closed referrals, in which the system verifies that the patient has made an appointment to see their clinician. The patient is reminded to make the appointment or the missed appointment. All users of the advice system are followed to verify the impact of the advice on clinicians’ prescriptions and patients’ depression-free days.

## 3. Results

### 3.1 Results for Component 1: Organization of the Knowledgebase 

Table 2 shows the cross-validated performance of LASSO regressions for predicting response to antidepressants. The area under the receiver operating characteristic curve (AUC), a measure of accuracy, ranged from 69.2% to 78.5%, indicating moderate predictive accuracy. The number of predictors that had a non-zero, robust coefficient in the LASSO regression ranged from 22 to 232 variables, indicating that response depended on many factors in the patient’s medical history. The number of unique medical history events needed to predict the responses to the 15 antidepressants was large and included 700 diagnoses, 550 current medications, 151 medical procedures, and 98 responses to previous antidepressants. Patients of AI-concordant clinicians were 17.5% more likely to experience a positive response to their antidepressant treatment than patients of AI-discordant clinicians.

**Table 2.** **Cross-validated accuracy of LASSO regression of response to medication**.

Antidepressant	Area under curve	Sensitivity	Specificity	Number of non-zero predictors
Amitriptyline	77.7%	21.0%	97.4%	40
Bupropion	74.0%	38.1%	96.0%	34
Citalopram	69.6%	63.1%	64.9%	173
Desvenlafaxine	74.6%	67.0%	69.8%	58
Doxepin	72.8%	48.8%	85.4%	38
Duloxetine	69.2%	55.7%	71.6%	129
Escitalopram	70.4%	42.7%	85.2%	108
Fluoxetine	70.6%	61.5%	67.9%	151
Mirtazapine	69.8%	37.6%	87.4%	44
Nortriptyline	72.3%	31.6%	92.7%	22
Paroxetine	69.9%	60.0%	68.9%	123
Sertraline	70.5%	64.1%	65.0%	195
Trazodone	78.5%	38.8%	98.1%	24
Venlafaxine	71.0%	64.8%	66.3%	138
Other	72.6%	55.1%	75.7%	232

LASSO, Least Absolute Shrinkage and Selection Operator.

Hughes *et al*. [41] also examined responses to 11 antidepressants using the continuation of medication as the outcome of interest, similar to our study. They relied on 10 predictors of response and reported an AUC that was lower than in our study. To improve the accuracy of the model, they selected 9256 medical history events that occurred for at least 50 patients. The study was limited to the use of antidepressants by psychiatric patients in two provider sites. The investigators concluded that response to specific medications cannot be anticipated from their predictive models. In contrast, our study relied on all patients, not just psychiatric patients. It was not restricted to a provider site and relied on insurance data nationwide. We used 40,784 medical history events, including prior response to use of antidepressants. In many of the models, positive experience with the antidepressant in the prior year was a key predictor of response to the same antidepressant for the next 10 weeks. In all models reported in Table 2, more medical history events were statistically significant than the total number of variables in the Hughes *et al*.’s study [41]. Our data suggest a moderate accuracy (AUC above 70% for most models) in predicting response to antidepressants.

### 3.2 Result for Component 2: Removing Algorithm Bias

Table 3 shows the predictive accuracy of models for predicting response to antidepressants among African American patients, as evaluated using the All of Us database. The comparison includes a general model and a population-specific model. The population-specific model was slightly more accurate in predicting responses to medications such as amitriptyline, fluoxetine, and trazodone compared with the general model approach. In predicting response to nortriptyline, the African-American-specific model explained 30% of the variation in response, while the general model explained 9% of variation, suggesting a large improvement in accuracy. The AI system was modified to use population-specific models for African Americans in predicting response to nortriptyline, amitriptyline, fluoxetine, and trazodone. For the 10 other antidepressants, the general models were more accurate than the population-specific models and therefore the general model was used to anticipate the responses of African Americans.

**Table 3.** **Accuracy of prediction of response to antidepressants among African Americans**.

	Amit	Bupr	Cita	Doxe	Dulo	Esci	Fluo	Mirt	Nort	Paro	Sert	Traz	Venl	Other
AD trials	1984	2658	2393	438	2590	2031	1984	1724	804	868	3596	4463	1448	523
Remission	780	1064	1036	138	1142	791	780	623	277	357	1497	1258	591	109
General model	5%	13%	23%	9%	13%	15%	5%	7%	9%	28%	17%	2%	22%	24%
Population-specific model	7%	12%	19%	12%	12%	19%	7%	9%	30%	16%	15%	1%	18%	16%

Notes: Desvenlafaxine was dropped from analysis because of less than 68 cases. AD, antidepressant; Amit, amitriptyline; Bupr, bupropion; Cita, citalopram; Doxe, doxepin; Dulo, duloxetine; Esci, escitalopram; Fluo, fluoxetine; Mirta, mirtazapine; Nort, nortriptyline; Paro, paroxetine; Sert, sertraline; Traz, trazodone; Venl, venlafaxine; Other, other less common antidepressants or combination of antidepressants. Accuracy is reported as McFadden’s R^2^.

### 3.3 Results for Component 3: Conversational Intake

A large-scale research project, funded by the Patient Centered Outcome Research Institute, is underway to test the ability of the intake process to stay on task, avoid hallucinations, and provide effective advice. Meanwhile, we tested the ability of conversational intake to recognize risk factors for suicide.

The AI system needs to clearly identify: (a) who is in, or likely to be in, an active suicidal crisis in the next few hours, (b) who is at sufficiently elevated risk of suicide for the system to alert the clinicians or the Safety Plan supporter, or (c) who is at a low enough risk of suicide that does not require additional actions from the AI system. The method of identification of patients who are in active suicidal crisis is the subject of significant research and it is not always clear how to do so [42]. One way is to directly ask the client: “Are you planning to kill yourself in the next few hours?” Patients may not answer this question, or other direct questions, about suicide truthfully. Another way is to infer from medical history events if the patient is at risk of suicide. This requires the AI system to analyze patient’s responses to medical history events and search for risk factors for suicide. For example, suicide risk might be increased if the client has painful diseases, sleep problems, or a history of self-harm. In conversations with the AI, the client may mention a variety of risk factors for suicide.

The accuracy of the LLM in detecting risk of suicide was examined in the analysis of 18 dialogues and 48 case descriptions found in two books, “The Suicidal Crisis” [43] and “Cognitive Therapy for Challenging Problems” [44], commonly used to train clinicians. The dependent variable in this analysis was a human rater’s classification of risk factors and the independent variable was the AI’s identification of the risk factors. On average the human rater and the LLM agreed in 97.03% (standard deviation of 3.98%) of risk factors in these dialogues/case descriptions. These data suggest that if the patient mentions risk factors for suicide, then the LLM can identify these risks.

Once the risk factors for suicide have been identified, the dialogue manager must still aggregate these risk factors and decide what to do next. There are numerous published indices for predicting risk of suicide from component risk factors [45]. None of these instruments are conversational and therefore it is not clear how they could be used during the intake conversation. One possibility is to use the count of risk factors to divert clients to different referral pathways: a crisis hotline, a Safety Plan supporter, or no referral for suicide risk. We examined how accurate the count of risk factors was in classifying the client’s risks. The dependent variable in this analysis was probability of suicide, as assessed by two experts in suicide detection. To assist the experts in assigning probabilities they made pair-wise comparisons of risks among different pairs of dialogues/case descriptions. If there were disagreements, a behavioral consensus was sought. The independent variable was the count of risk factors identified by the AI system. The count of risk factors explained 30% of the variation in experts’ rating of the 66 dialogues and case descriptions. When the count exceeded three risk factors, then it nearly perfectly (AUC 99%) classified the dialogues and case descriptions into low versus high/moderate risk groups. These data suggest that the count of risk factors may be a reasonable way to make referrals to different levels of care for suicidal patients. Further research is needed to clarify if count of risk factors is sufficient.

It is important to point out that the sample size of 66 dialogues/case descriptions is small. A larger sample is needed to further validate our findings. We took the dialogues/case descriptions from books used to train clinicians. These dialogues/case descriptions may not be representative of the types of conversations that occur in real life, which are typically far more ambiguous. Real conversations may be less grammatically correct. There might be more misspelled words. In a conversation with an AI system, patients may be less forthcoming if they feel the machine is not empathetic. They may be more forthcoming if they feel that the machine is less likely to judge them. We have compared AI performance to experts’ opinions. In doing so, we have assumed that the experts’ consensus is the gold standard that needs to be replicated by the machine. In suicide risk assessment, even experts could be inaccurate. Thus, the comparison of AI’s performance with experts may not be reasonable. These limitations suggest that additional studies are needed to be carried out before we are reassured about the ability of an AI system to monitor suicide risks.

### 3.4 Results for Component 4: Dialogue Management System

A minimum viable prototype for the survey method is available at http://MeAgainMeds.com. A working viable prototype of the conversational intake is available at http://rapidimprovement.ai. This prototype includes the use of dialogue management to keep patients on task.

### 3.5 Result for Component 5: Human-in-the-Loop Monitor

This component has not yet been implemented.

### 3.6 Results for Component 6: Advice System

The AI system used the equations within its knowledgebase to predict responses to antidepressants. Because there was a mismatch between patient reported medical history events and the list of events needed for predicting responses, the study imputed the missing values from 1499 predictors of responses to antidepressants. The Python code for these imputations is available upon request from the first author of this article.

Once the probability of response for each of the common oral antidepressants was calculated, the Python code prepared the text of the advice to the client. The advice had the following structure:

(1) **Summary of the client’s medical history**: The advice system would summarize what the patient had said to the intake system and list items in the medical history that were relevant to the choice of antidepressants.

(2) **Summary of the advice**: This section of the advice describes which antidepressant is likely to have the highest response rate. If the client does not have moderate or severe depression, no antidepressant is recommended and the client is referred to known treatment for low severity depression, including exercise. If the client has moderate to severe depression and if at least one of the antidepressants increases the response rate by more than 10%, then the system recommends the antidepressant with the highest predicted response rate. If no antidepressant has at least a 10% chance of response, the system will recommend that the patient rely on other treatment options besides common oral antidepressants. If the top two antidepressants had response rates that were within 5% of each other, then the system would recommend the use of either one of the two antidepressants. The system also produces a bar chart showing the response across all antidepressants.

(3) **Explanation of the advice**: The system lists medical history events that were not reported but the presence of these events would change the recommendation of the system. It also lists reported events that, if they were absent, would change the recommendation of the system. These steps are taken to highlight how a change in medical history could change the advice of the system.

(4) **More information**: The system provides a link for the recommended antidepressant where the client can examine the side-effects and other research on this medication.

These components of the advice system are available at the web pages described in the Results for Component 4 section. In these implementations, the advice system for the survey method was based on factors with a large impact on the response rate. It ignored rare combinations of factors that occurred in less than 100 out of more than 3 million patients examined. The advice system for the conversational intake included all relevant medical history events but assumed that events not mentioned have not occurred. At the time of publication of this report, the advice system did not take advantage of the joint distribution of events to impute missing values.

### 3.7 Results for Component 7: Coordination of Care

This component of the system is under construction.

### 3.8 Results for Cross Component Findings

**Patient’s need for the Artificial Intelligence system**: We relied on an online advertisement to reach patients because more than half of patients with MDD are no longer in treatment and cannot be reached through clinic recruitment [46]. We advertised on Google and Facebook for 2 weeks to assess the demand for the decision aid. On Google, we advertised to 29,636 individuals in Virginia. The daily rate of recruitment was 15.25 individuals per day. On Facebook, we advertised to 50,501 individuals in Virginia. The daily rate of recruitment was 39.64 individuals per day. The project successfully advertised the availability of the web site to 80,137 Virginia residents and 1536 depressed Virginia residents used the system. The number who completed the survey decision aid or who found the information useful is not known. At the time of evaluating this component of the AI system, to protect the patient’s privacy and encourage use of the system, information on use of the system and patient identifiers were not collected. The only information kept was the number of unique individuals accessing the system. These data show that patients were interested in receiving advice from the AI system. Because patient data were not kept (patients were classified into one of 16,775 prototype categories based on their responses), the Institutional Review Board declared that the study was exempt from review. Later versions of the system, which collected patient identifiers and kept patient responses, did require consent and ethical review.

**Clinicians’ attitudes towards the AI system**: In a series of interviews and focus groups, our colleagues assessed the reaction of 29 psychiatric and primary care providers to the proposed decision aid. Several positive and negative points were raised. Clinicians said that “Prospective random trials are needed”. Clinicians claimed that the aid did not include “the type of patients I see” or “In my clinic, patients have different types of depression”. They said that “Prescribing antidepressants is an art and not science and involves negotiating many issues with the patient”. Some said that they are different from other clinicians and more “careful about antidepressant prescriptions”. Many clinicians were surprised that we were not following consensus guidelines. One clinician who carefully examined our advice system pointed out that the AI lists “eye problems as a condition for altering antidepressants”, which in his experience was not credible, even though a published study supported the AI’s advice [47].

Because the AI system recruits patients online and advises them prior to the clinic visit, the process could generate new referrals. We contacted Psychiatric Mental Health Nurse Partitioners (PMHNPs) primarily in Virginia and Maryland through the LinkedIn platform. The mental health clinicians contacted were willing to receive referrals from the AI system. Of particular interest were rural PMHNPs, who saw the referral as a method of expanding their telemedicine services to a wider catchment area, which could include the entire state, including urban areas. Twenty clinics were willing to receive referrals and discuss the advice of the system with the patients.

## 4. Discussion

Herein, we described the development, initial testing, and availability of an AI-driven decision aid designed to optimize antidepressant prescriptions. The system, which leverages patient-specific data, aims to enhance treatment outcomes through personalized recommendations. Although freely accessible online, further testing and improvements are ongoing. The initial data indicates that response to antidepressants is predictable with moderate levels of accuracy from patients’ medical history. These data also show that many medical history events are relevant to anticipate a response to antidepressants. The size of the relevant medical history has encouraged the use of conversational intake instead of multiple-choice survey methods. Initial experimentation with the system has established a working prototype and has shown that patients are interested in receiving its advice. Furthermore, clinicians are willing to receive referrals from it.

Investigators need to carefully examine the two methods for improving patient safety: AI and human-in-the-loop suicide risk assessment and management. We reported that human and machine agreed on 97% of suicide risk factors within simulated dialogues. This level of agreement suggests that machines might be able to detect suicidality in patients’ interactions with the LLM. Whether this is sufficient is not clear. It is also not clear if human monitoring of the conversations is efficient and effective. Data show that experienced clinicians are often inaccurate in assessing the suicide risks of patients, particularly at discharge from psychiatric hospitals [48]. Analytical models also have a high number of false alarms [49]. It is not that models are better, or worse, than human monitors, neither the machine nor the human is a good predictor of suicides. Future studies need to address whether the combined human-in-the-loop and AI system is sufficiently accurate to create a safe environment for depressed patients to interact with AI.

A pragmatic, retrospective study was conducted to validate and refine the AI system’s knowledgebase. The system’s accuracy was tested using large-scale data from OptumLabs to ensure its predictions were reliable across diverse patient populations. This is a test in a single but massive database. With minor exceptions, similar accuracy levels were obtained from the All of Us databases. These cross-database evaluations of the AI’s knowledgebase suggests that findings are stable and not an artifact of the data.

A study is needed to examine how to address missing values and unreported events. Analytical language and LLMs differ in how they address missing values. In an analytical model, the formula works only if all variables are specified. When one variable is missing, the entire formula cannot be used, and the missing value must be imputed. Often, unreported values are assumed to be absent. Language does not work in the same way. Language models do not require all relevant values to be specified, before giving advice. One way to make the AI’s input more robust is to build in missing value imputation models. Instead of using regression, one could use a network model that includes both the initial regression predicting response to the antidepressant and additional imputation models that are useful when predictors of response are missing [50]. A network model estimates missing values from prior available information. This takes into account the joint distribution of the data and may be more accurate than assuming that unreported events have not occurred. A study is needed to examine if imputing missing values will increase the stability of AI’s advice and prevent model drift and deterioration in new applications.

It is important to further examine the algorithmic bias. We evaluated the accuracy of predictive models for response to antidepressants in African American patients. These findings underscore the importance of addressing disparities in treatment recommendations and updating the AI’s knowledgebase accordingly. Herein, we reported our experience with African American patients and additional research is needed to adjust the AI system for Hispanics and other subgroups of patients with MDD.

A prospective randomized clinical random trial is needed to examine if the adoption of the AI-guided care by clinicians and its impact on patients’ outcomes. A study of impact of adoption of AI care is not the same as a study evaluating the accuracy of the knowledgebase of the AI system. A prospective clinical trial can address if clinicians will use AI guided care. Because of its narrow inclusion/exclusion trial it cannot address if the vast knowledgebase of the AI system is accurate. Therefore, in addition to prospective random clinical trials we call for continued database observational studies that can clarify the knowledgebase of the AI system. A pragmatic database study could perform a better and more detailed evaluation of various aspects of the AI’s knowledgebase.

A prospective, random trial could address the impact of the AI on the clinician’s practice patterns. Some clinicians will not follow the system’s advice and others will. The study could clarify if patients of AI-concordant clinicians experience better outcomes. In particular, the study could address whether these patients have more depression free days. Since a portion of the clinicians may not follow the system’s advice, the study needs to simulate what would have happened if they did, i.e., the study needs to estimate a counterfactual likelihood of the unrealized impact of the system.

Should an AI system be deployed now? — Despite the need for additional studies, a question remains about what clinicians should do until such studies are available. “Only 22% to 40% of the patients benefit from their antidepressants” [51]. The current situation is not tolerable. Clinicians have a choice. They can wait for more information or start using the aid as a supplementary tool. The clinician may ask for advice from the aid but discard it. There is, however, a chance that they may do better if the AI system enables them to tailor prescriptions to patients’ medical history. Given the current status of treatment of MDD, some clinicians might think that this is a chance worth taking.

Use of AI system to train clinicians — One way that clinicians can think about AI is as feedback from patient experiences across many patients, beyond their own practice. This feedback is currently not available. Depression is both an indicator and a barrier to treatment [52]; many patients do not return for adjustment of their antidepressants. Even when patients return, clinicians often cannot decipher patterns across their own patients without careful statistical study. Typically, clinicians have only selective and anecdotal feedback on whether their prescriptions are working. The AI system can help clinicians, especially those in training, to access a new source of feedback that is broader than their experiences with their own patients. Relying on this feedback may help clinicians to gain new insights.

Relationship with consensus guidelines — Finally, we acknowledge that the proposed AI system does not follow consensus guidelines. These guidelines prescribe antidepressants based on the “serotonin hypothesis”. This hypothesis encourages clinicians to first prescribe antidepressants that work directly with serotonin reuptake inhibition; other medications are introduced if the first-order antidepressants fail. Many have raised doubt about the serotonin hypothesis and have suggested alternative mechanisms of action, in particular chronic stress [53]. The stress mechanism allows for a variety of comorbidities, from cancers to sleep disturbances, to affect response to antidepressants. The system presented here weighs these comorbidities in anticipating response to antidepressants, while consensus guidelines do not. Therefore, differences in recommendations are expected. Until more information is available, clinicians must decide which is best for their patients: consensus guidelines or an evidence-based intake and recommendation from an AI system.

## 5. Conclusions

This manuscript outlines the initial development, progress, and future research directions for an autonomous, AI-driven decision aid designed to optimize antidepressant management for MDD. The AI system integrates predictive analytics, a conversational LLM, algorithmic bias mitigation strategies, dialogue management, human-in-the-loop monitoring, personalized treatment advice, and clinician care coordination to improve antidepressant effectiveness. Preliminary evaluations demonstrate that the patients of clinicians who prescribe consistent with the advice of the system are more likely to experience remission than patients of clinicians who do not follow the advice of the system. The system also shows promising capabilities in recognizing suicide risk factors through conversational intake, underscoring its potential to contribute to patient safety. Future research priorities include validating the system’s efficacy and safety through prospective randomized trials, refining algorithmic bias adjustments across diverse patient populations, and improving the robustness of conversational interactions. Clinicians may consider the current AI system as a supplementary tool, facilitating personalized and evidence-based antidepressant prescriptions.

## Data Availability

The antidepressant knowledgebase of regression coefficients is available in the appendix of our prior work, “Assessment of a Prediction Model for Antidepressant Treatment Stability Using Supervised Topic Models” (https://doi.org/10.1001/jamanetworkopen.2020.5308).
